# The Genetic Side of the Mood: A Scientometric Review of the Genetic Basis of Mood Disorders

**DOI:** 10.3390/genes14020352

**Published:** 2023-01-30

**Authors:** Giovanni Bonacina, Alessandro Carollo, Gianluca Esposito

**Affiliations:** Department of Psychology and Cognitive Science, University of Trento, Corso Angelo Bettini 31, 38068 Rovereto, Italy

**Keywords:** mood disorders, genetics, scientometrics, document co-citation analysis, affective disorders, bibliometrics

## Abstract

Mood disorders are highly heritable psychiatric disorders. Over the years, many genetic polymorphisms have been identified to pose a higher risk for the development of mood disorders. To overview the literature on the genetics of mood disorders, a scientometric analysis was performed on a sample of 5342 documents downloaded from Scopus. The most active countries and the most impactful documents in the field were identified. Furthermore, a total of 13 main thematic clusters emerged in the literature. From the qualitative inspection of clusters, it emerged that the research interest moved from a monogenic to a polygenic risk framework. Researchers have moved from the study of single genes in the early 1990s to conducting genome-wide association studies around 2015. In this way, genetic overlaps between mood disorders and other psychiatric conditions emerged too. Furthermore, around the 2010s, the interaction between genes and environmental factors emerged as pivotal in understanding the risk for mood disorders. The inspection of thematic clusters provides a valuable insight into the past and recent trends of research in the genetics of mood disorders and sheds light onto future lines of research.

## 1. Introduction

Mood disorders are a group of psychiatric disorders characterized by a severe impairment of mood which lasts for a prolonged period of time. The intensity of mood disorders typically compromises interpersonal relationships and working performances [1]. Mood disorders are described in terms of depression (i.e., extreme emotional lows [2]) and mania (i.e., extreme emotional highs [3]), which are regarded as the extremes of the mood *continuum* [4]. In the fifth edition of the Diagnostic and Statistical Manual of Mental Disorders (DSM-5), the group of mood disorders is divided into unipolar and bipolar disorders [5]. On the one hand, unipolar disorders refer to psychiatric conditions characterized only by depressive episodes. On the other hand, bipolar disorders refer to oscillations between depressive and manic mood states.

In line with the bio-psycho-social model [6], the complex etiology of mood disorders is understood by considering the interaction between biological, psychological, and social factors. Particularly, genetics seem to play a significant role in mood disorders. Decades of family studies have provided evidence of a shared familial risk for the development of mood disorders [7]. Specifically, twin and adoption studies indicate the strong contribution of genetic factors in the risk for these psychiatric disorders [8,9]. In fact, genetic factors seem to explain about 35–45% of variance in the etiology of major depression disorder and 65–70% of variance for bipolar disorder [9,10]. In recent years, the identification of genetic variations that increase the susceptibility for mood disorders has benefited from the introduction of genome-wide associations studies (GWAS) [10,11,12]. From recent studies, it emerged that common genetic variations account for 9–23% of the clinical phenotypic variation in mood disorders [8,10,11]. Conversely, rare genetic variations contribute less to the development of mood disorders as compared with other psychiatric disorders [8].

A plethora of genetic loci emerged from the genetic literature on mood disorders. The current work aims to provide an updated and data-driven review to summarise the main scientific contributions in this prolific discipline. The focus of the study is to identify and discuss the thematic domains that shaped the genetic research on mood disorders, key publications, and the most active countries. In doing so, the current study aims to identify gaps and future directions of research. To achieve these objectives, the scientometric approach to systematic reviews was used [13]. Scientometric analysis is rooted on the intersection between scientific mapping (i.e., the visual inspection of the temporal evolution of a research domain) and bibliometric analysis [14,15]. Scientometric reviews are a type of data-driven reviews, which allow to map fields of knowledge using quantitative relationships between publications [16]. Not only the scientometric approach allows to summarize a topic of interest. Rather, as discussed by Sabe et al. [15], it provides insight into common thematic domains, research gaps, potential moderators, bias, or study limitations. Scientometrics is gaining *momentum* and it has proved useful in medicine, clinical psychology, social and molecular neuroscience (e.g., [15,17,18,19,20]).

## 2. Materials and Methods

### 2.1. Data Collection

The current review follows the overall procedure of previously published scientometric works in the field of neuroscience and psychology (see, for instance, [17,18,19,20,21,22,23]).

Data for the current review were collected from Scopus on 13 December 2022. Scopus was preferred to other platforms (e.g., PubMed, PsycINFO) for its wide coverage of indexed journals and documents [20,24,25]. The following string was used: “TITLE-ABS-KEY ((genotype OR genetics OR genome) AND (“mood disorder*” OR “affective disorder*”)) AND (LIMIT-TO (LANGUAGE, “English”))”. The string of keywords was designed to collect a wide and precise sample of publications on the genetic research in mood disorders. The language was limited to English in order to include only the documents meeting the international standards for publications [18,26]. When performing the literature search, the authors conducted a visual inspection of documents’ titles to ensure that the identified publications were relevant for the genetic research in mood disorders. Terms used in the research string were optimized to reduce noise in the sample of retrieved publications. Furthermore, no time criterion was used to limit the research. In this way, a sample of 5342 scientific documents published between 1965 to 2023 were identified and downloaded. Of note, the online version of some documents *in press* for 2023 was already available on Scopus. For this reason, these documents were included in the analysis.

### 2.2. Data Import in CiteSpace and Eligibility of References

The set of downloaded publications was imported in CiteSpace (version 6.1.R2) [27], the software used to conduct the scientometric analysis. CiteSpace identified an amount of 191,211 references cited by the documents downloaded from Scopus. Of the total references, an amount of 189,211 was considered valid (data loss rate = 1.05%). Similar negligible rates of data loss, which typically range from 1.00% to 5.00%, are caused by irregularities in the citation format [27,28]. Subsequently, the “Remove Alias” function was adopted to eliminate repeated or identical entries in the data pool [20,23].

### 2.3. Document Co-Citation Analysis

To identify key publications and main thematic domains in the literature on the genetics of mood disorders, a document co-citation analysis (DCA) was performed on CiteSpace [29,30]. DCA is a bibliometric analysis based on peer recognition in terms of citations. In fact, DCA measures the frequency in which two or more documents are co-cited (i.e., cited together) by subsequent publications [31,32]. Frequent co-citations among documents are considered a bibliometric marker for a shared thematic domain [19,33]. From the patterns of co-citation, the DCA allows to generate a network which includes two main types of documents: citing and cited documents. DCA generates a network based on the type of criteria used to select the network’s nodes and on the set threshold, namely, the scale factor [24]. In CiteSpace, there are three main node selection criteria: g-index, TOP *N* and TOP *N%*. The g-index is an implementation of the h-index and it could be defined as “largest number that equals the average number of citations of the most highly cited g publications” [34,35,36]. Overall, as compared to the h-index, g-index has the advantage of mitigating bias due to an author’s highly cited papers as it takes into account also poorly cited documents. This characteristic causes the g-index to be the optimal parameter for co-citation studies [15]. Differently, the TOP *N* and TOP *N%* node selection criteria allow to include in the final network the *N* or *N%* most cited documents in a selected period of time (i.e., time slice) [37]. In the present study, time slice was always maintained fixed at the value of 1 year to maximise the amount of retrieved information. As done in previous scientometric reviews (see for instance [22]), the following node selection criteria were tested and compared to optimise the final results: g-index with *k* set at 15 and 25, TOP *N* with *N* set at 25 and 50, and TOP *N%* with *N* set at 15. The overall effects of the selected parameters on the network’s structural properties (i.e., number of nodes, number of links, number of clusters, modularity, silhouette, and harmonic mean) was compared. G-index with *k* set at 25 emerged as the optimal parameter to conduct the DCA and generate the network of documents.

**Figure 1:**
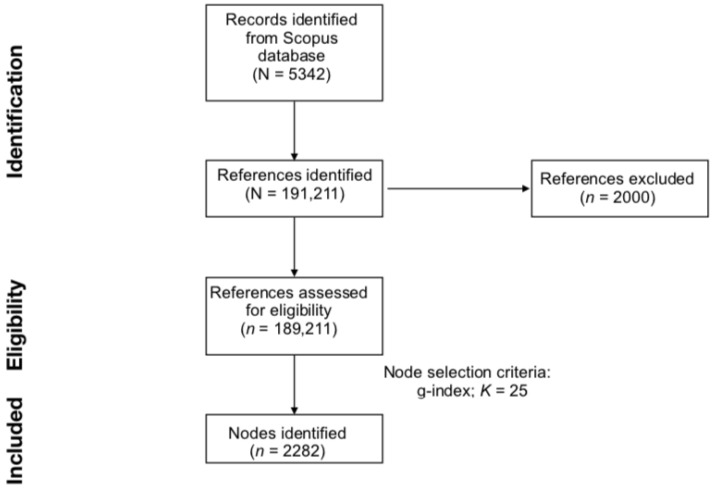
Preferred Reporting Items for Systematic Reviews and Meta-Analyses (PRISMA) flowchart for literature search and reference eligibility.

The literature search and the generation of the DCA network are summarised in Figure 1.

### 2.4. Country Analysis

To identify the most active countries in the genetic research of mood disorders, we performed a country analysis using g-index with *k* set at 25.

### 2.5. Metrics of Interest in CiteSpace

CiteSpace’s DCA generates results in terms of structural and temporal metrics [17].

Structural metrics includes *modularity Q*, *silhouette score*, and *between centrality*. Modularity Q provides information about the degree for which the network is divisible into single modules or clusters [38]. Modularity ranges between 0 and 1, where the higher the value and the more divisible and structured the network is [33]. Conversely, silhouette score indicates the internal consistency of a single cluster and in its degree of separation from the other clusters in the network [39]. Its values range between −1 and 1, with 1 representing highly homogeneous clusters that are highly separated from the rest of the network [40]. Betweenness centrality measures the degree in which each single node functions as a bridge in connecting two other arbitrary nodes in the network [41,42]. Betweenness centrality values range from 0 to 1, with greater values indicating the most far-reaching and revolutionary works [19,40].

Temporal metrics include *citation burstness* and *sigma*. Citation burstness is computed by means of the Kleinberg’s algorithm [43] and it is a measure of any abrupt increase in the number of citation received by a specific document over a period of time. Citation burtsness allows to identify publications that have received ample acknowledgment and attention from the scientific community [44]. Values for citation burstness range from 0 to infinity. Finally, sigma indicates the degree of novelty and impact of a specific document within the field of interest [13]. Sigma values are computed by combining both between centrality and citation burstness through the equation (centrality + 1)${}^{burstness}$ [33].

## 3. Results

### 3.1. Structural Properties of the DCA Network

**Figure 2:**
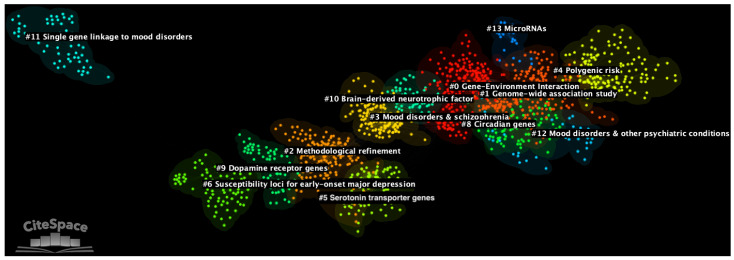
Document co-citation analysis network of all literature on the genetics of mood disorders with 13 generated clusters. The earliest thematic clusters appear on the left of the figure, while more recent clusters are displayed on the right. In the network, single nodes represent individual documents. The image was generated with the CiteSpace software [27].

The final network of documents generated by means of DCA included a total of 2282 nodes and 8951 links (Figure 2). On average, each node had 3.92 connections. The final network had modularity Q equalling 0.8127 and weighted mean silhouette of 0.9132. Thus, the generated network could be largely divided into highly homogeneous and consistent thematic clusters.

In the network, 13 major thematic clusters were identified (see Table 1). Clusters were automatically labelled using CiteSpace’s Log-Likelihood Ratio (LLR) algorithm [37]. LLR was chosen because it usually provides the most accurate automatic labels as compared with other methods [28]. However, the accuracy of clusters’ labels was ascertained by the authors with a qualitative inspection of documents’ titles and abstracts. When the automatically generated labels were lacking accuracy, clusters were manually renamed based on the common thematic interest of the documents, as in previously published scientometric reviews [17,19]. In terms of the number of included documents—in particular, their size—the main clusters were: cluster #0 with 241 documents (silhouette = 0.895; average year of publication = 2010), cluster #1 with 232 documents (silhouette = 0.834; average year of publication = 2015), and cluster #2 with 173 documents (silhouette = 0.907; average year of publication = 1999). In terms of silhouette score, the clusters with highest internal consistency were: cluster #13 with silhouette equalling to 0.989 (size = 28; average year of publication = 2015), cluster #11 with silhouette equalling 0.987 (size = 56; average year of publication = 1991), and cluster #12 with silhouette equalling to 0.984 (size = 37; average year of publication = 2013). Cluster #4 (size = 149; silhouette = 0.941) was the most recent cluster in the network, consisting of documents that on average were published in 2020. In terms of recency, cluster #4 was followed by cluster #13 (average year of publication = 2015; size = 28; silhouette = 0.989) and cluster #1 (average year of publication = 2015; size = 232; silhouette = 0.834). Finally, the three earliest clusters on the genetics of mood disorders were cluster #11 (average year of publications = 1991; size = 56; silhouette = 0.987), cluster #6 (average year of publication = 1994; size = 68; silhouette = 0.979), and cluster #9 (average year of publication = 1997; size = 85; silhouette = 0.973).

### 3.2. Impactful Documents

In the network, a total of 308 documents showed a citation burst in their citation history (see Table 2). In particular, the longitudinal study by Caspi et al. [45] was the document with the strongest citation burst, with a value of 41.14 (burst duration = 7 years; sigma = 14.26). In their impactful work, Caspi et al. [45] investigated the circumstances for which life stress leads to depression in some cases but not in others. The authors observed that a polymorphism in the promoter region of the serotonin transporter gene plays a moderating role in the relationship between life stress and depression, with individuals carrying at least one short allele that exhibit higher depressive symptoms. The work by Caspi et al. [45] was one of the first studies to adopt a gene-by-environment framework to uncover the underlying mechanisms of a psychiatric disorder. In terms of burst strength, Caspi et al. [45] was followed by Pantelis et al. [46], who obtained a citation burstness value of 29.67 (burst duration = 8 years; sigma = 14.08) and by a Barrett et al. [47] (strength of burst = 27.31; burst duration = 5 years; sigma = 1.16). In the network, Pantelis et al. [46] presented the longest burst duration with a value of 8 years, followed by Caspi et al. [45] (burst duration = 7 years) and Stine et al. [48] (burst duration = 7 years; burst strength = 25.42; sigma = 1.38). Caspi et al. [45] also obtained the greatest value of sigma, with a value of 14.26, followed by Pantelis et al. [46] (sigma = 14.08) and by Purcell et al. [49] (sigma = 7.30; burst strength = 22.04; burst duration = 5 years). These documents with high sigma represent innovative and impactful publications in the genetic research of mood disorders.

### 3.3. Most Active Countries

The network generated for the country analysis consisted of 187 nodes (i.e., countries) and 1175 links. On average, each node was connected to another 6.28 nodes. The most frequent countries that appeared in the network were the United States of America (count = 2226). The United States of America were followed by the United Kingdom (count = 703), Germany (count = 624), and Italy (count = 436).

A total of 14 countries had a citation burst in their history. The countries with the highest citation burst were the United States of America (burst strength = 49.97; burst duration = 20; sigma = 32,366.55), Belgium (burst strength = 12.54; burst duration = 8; sigma = 1.30), and China (burst strength = 11.92; burst duration = 7; sigma = 1.53).

## 4. Discussion

In the following section, the scientific content of the identified major thematic clusters will be discussed. Clusters will be presented following the chronological order in which on average their documents were published. When discussing the clusters, the main citing documents will be presented together with their coverage (i.e., number of documents in the cluster that were cited by the paper) and Global Citation Score (GCS; i.e., the total number of citations received by the publication on Scopus) [20].

### 4.1. Cluster #11: Single Gene Linkage to Mood Disorders

Cluster #11 included documents that were published, on average, in 1991. The major citing documents of the cluster were authored by Hill et al. [55] (coverage = 8; GCS = 13), and Le et al. [56] (coverage = 6; GCS = 30). In their work, Hill et al. [55] propose linkage analysis as a method to discern between genetics and environmental influences. In this way, Hill et al. [55] identified three possible genetic markers of mood disorders: GYPA gene, Orosomucoid 1 and GC Vitamin D Binding Protein. In a similar vein, some works included in the cluster highlighted the genetic linkage between mood disorders, especially bipolar disorders, and DNA markers in chromosome 11p15 [57] or in chromosome X [58]. However, contradictory results were observed in subsequent studies included in the cluster (e.g., [59]). In the cluster, particular attention was given to investigating the risk posed by dopamine receptors genes (i.e., DRD1 [60], DRD2, DRD3, and DRD4 [61]) in the development of mood disorders. Significantly, the linkage study by Nanko et al. [61] observed that only linkage of mood disorders with DRD4 could not be excluded. In summary, the cluster included documents in which the initial relationship between the genetic underpinnings of mood disorders was conducted. For this thematic interest, the cluster was manually renamed as “Single gene linkage to mood disorders”.

### 4.2. Cluster #6: Susceptibility Loci for Early-Onset Major Depression

Cluster #6 consists of documents published on average in 1991. The major citing documents in the cluster were authored by Zubenko et al. [62] (coverage = 13; GCS = 58), Zubenko et al. [63] (coverage = 11; GCS = 98) and Zubenko et al. [64] (coverage = 10; GCS = 34). As the name of the cluster suggests, the majority of the included documents focused on identifying the genetic loci underlying recurrent early-onset major depressive disorder. In fact, the major citing documents suggested that some genetic regions (e.g., CREB1 gene) show a significant correlation with mood disorders [62,63,64]. Curiously, the correlations between the identified genetic regions and mood disorders were stronger in woman participants. This observation might explain the higher prevalence of mood disorders in women as compared to men [65]. In the cluster, some documents continued focusing on the single gene risk for mood disorders with ambiguous results. For instance, DRD5 emerged as not being involved in the development of mood disorders [66] and pedigree studies on the linkage between mood disorders and genetic markers in chromosome 11 did not show significant results [67]. However, the study by Blackwood et al. [68] identified that the genetic marker D4S394 in chromosome 4p is associated with bipolar disorders [68]. Similarly, marker D2S44 of the chromosome 2q21 region seems to be related to mood disorders [69].

### 4.3. Cluster #9: Dopamine Receptor Genes

Cluster #9 is a group of documents published, on average, in 1997. The major citing documents in cluster #9 were authored by Serretti et al. [70,71] and Turecki et al. [72]. Specifically, Serretti et al. [70] reported a coverage of 14 and GCS of 7, Serretti et al. [71] had coverage of 13 and GSC of 36, and Turecki et al. [72] had coverage of 12 and GSC of 42. The documents included in cluster #9 focus mainly on the role played by dopamine receptor genes in the pathogenesis of mood disorders. For instance, Serretti et al. [71] observed that dopamine receptor D4 gene is associated with delusional symptoms in mood disorders. The link between dopamine receptor D4 gene and delusions in mood disorders was also supported by further works [73,74]. Similarly, in the cluster, dopamine receptor genes were investigated in relation to specific sub-elements of mood disorders (e.g., delusions, lithium prophylaxis, manic symptoms) [72,74,75]. Interestingly, dopamine receptors D2 and D4 genes did not appear to be involved in the individual’s susceptibility to lithium treatment, even when the sample was stratified by gender, polarity, family history, onset and duration of treatment [75].

### 4.4. Cluster #2: Methodological Refinement

Cluster #2 includes documents published, on average, in 1999. In cluster #2, the major citing documents were authored by Potash and DePaulo Jr [76] (coverage = 49; GCS = 73), Johansson et al. [77] (coverage = 22; GCS = 27), and Oswald et al. [78] (coverage= 21; GCS = 7). Cluster #2 was manually renamed as “Methodological refinement” as the included documents oftentimes addressed common methodological issues in the genetic research in mood and psychiatric disorders. In the cluster, some documents focused on the type of population and sample size to recruit in genetic studies [76,78,79]. Several documents discussed the usefulness of creating symptom or physiology-based (and not diagnosis-based) groups to investigate the genetic mechanisms of psychiatric disorders and to improve the reliability of findings between different genetic studies [76]. Accordingly, many scholars directed their effort in defining putative endophenotypes [80]. Endophenotypes represent more basic phenomena and their variability is assumed to be explained through a lower number of genes as compared to the macro-phenomena used for diagnosis in psychiatry [80,81]. In parallel, statistical and technical advancements (e.g., non-parametric methods, microarrays, genome-wide associations studies) provided new instruments to investigate the genetics of mood disorders [76,77,78]. For instance, to tackle the scarce replicability of linkage studies, the impactful document by Berrettini et al. [50] used non-parametric methods to investigate genetic susceptibility for bipolar disorders. By doing so, the authors identified a candidate genetic marker for bipolar disorder in chromosome 18. This finding was supported by Stine et al. [48], which was one of the most impactful documents in the network.

### 4.5. Cluster #5: Serotonin Transporter Genes

In cluster #5, documents were published, on average, in 2001. The major citing document of the cluster is a review by Serretti et al. [82], with coverage of 23 and GCS of 69, on pharmacogenetics in mood disorders. Other major citing documents were authored by Anguelova et al. [83] (coverage = 20; GCS = 253) and by Serretti et al. [84] (coverage = 18; GCS = 167). The publications included in the cluster investigated the role of serotonin transporter and receptor genes in the etiology, symptomatology (e.g., suicidal behaviour) and treatment of mood disorders (e.g., [83,85]). This group of works took inspiration from research previously conducted on anxiety. The impactful document by Lesch et al. [52] was strongly cited in the cluster. This study reported an association between the short variant of the polymorphism for the serotonin transporter gene and personality traits related to anxiety. In the context of mood disorders, Serretti et al. [82,84] documented that specific polymorphisms in the promoter region of the serotonin transporter gene (SL6A4) influence both the effectiveness of acute and long-term treatment by means of anti-depressant and lithium, respectively. In the cluster, some initial studies observed some commonalities in terms of serotonin transporter and receptor genes between psychiatric conditions, such as mood disorders and substance abuse disorder [86], and mood disorders, neurodevelopmental (e.g., autistic disorder) and neurodegenerative (e.g., Alzheimer’s disease) disorders [87].

### 4.6. Cluster #3: Mood Disorders and Schizophrenia

On average, the documents included in cluster #3 were published in 2005. In cluster #3, the major citing documents were authored by Craddock and Forty [7] (coverage = 36; GCS = 184), Potash [88] (coverage = 35; GCS = 74), and Bogaert et al. [89] (coverage = 30; GCS = 14). Craddock and Forty [7] highlighted the complexity of clinical phenotypes in terms of overlap between diagnostic categories. Accordingly, many studies included in the cluster documented strong genetic commonalities between mood disorders and schizophrenia (e.g., [88,89,90,91]). As the hypothesis of a common genetic signature between mood disorders and schizophrenia emerged, criteria for the classification of clinical phenotypes began to be questioned too. In fact, the distinction between schizophrenia and mood disorders, especially bipolar disorders, became more nuanced and led to the conceptualization of schizoaffective disorder [88]. The notion of schizoaffective disorder was not new. In fact, while in his work Kreaplin clearly distinguished between psychotic and mood disorders, some overlap of these nosological categories was already described in the first half of the 20th century [92]. Particularly, Kasanin [93] reported the case of nine young patients in which manic and/or depressive symptoms were intertwined with psychotic episodes and introduced the term “schizoaffective psychosis” [92].

### 4.7. Cluster #10: Brain-Derived Neurotrophic Factor

Documents included in cluster #10 were published, on average, in 2006. In cluster #10, the major citing documents were authored by Fan and Sklar [94] (coverage = 21; GCS = 41), Tsai and Hong [95] (coverage = 20; GCS = 1), and Liu et al. [96] (coverage = 18; GCS = 48). Publications in the cluster focused mainly on brain-derived neurotrophic factor (BDNF) gene and on its role in the pathogenesys of mood disorders. One of the first documents to report a possible association between BDNF and mood disorders was authored by Sklar et al. [53]. This document was identified as one of the most impactful in the genetic research of mood disorders. Subsequently, Liu et al. [96] showed how variations in BDNF gene (especially the single nucleotide polymorphism *Val66Met*) are related to the development of mood disorders and to their clinical phenotype (e.g., frequency of depressive/manic episodes). These conclusions were corroborated by subsequent studies [94,95]. In fact, BDNF gene has a strong impact on neurodevelopment, as it appears to modulate neuronal growth, development, differentiation, and survival [95]. BDNF gene interacts with other genetic regions modulating the levels of neurotransmitters (e.g., serotonin-transporter-linked promoter region and dopamine transporter gene) in the pathogenesis of mood disorders. On the one side, the genotype on the *Val66Met* polymorphism alone is associated with higher neural responses to emotional stimuli in the right amygdala [97]. On the other side, when the combination of *Val66Met* and a specific variation of the dopamine transporter gene (i.e., absence of the 9-repeat allele) is associated with neuroticism [98]. This is important as neuroticism is a personality trait that is regarded as a risk factor for many psychiatric disorders, especially for mood disorders. Finally, in the cluster, BDNF gene was explored also in terms of lithium treatment efficacy [95,99]. From the study by Rybakowski et al. [99], the genotype on the *Val66Met* polymorphism was associated with the quality of lithium prophylaxis.

### 4.8. Cluster #0: Gene–Environment Interaction

Cluster #0 is a group of documents published, on average, in 2010. The major citing works in the cluster were authored by Bogaert et al. [89] (coverage = 38; GCS = 38), Mann and Currier [100] (coverage = 29; GCS = 19), and Kato [90] (coverage = 28; GCS = 237). In particular, the chapter by Mann and Currier [100] highlighted that there are individual differences in the vulnerability to stressful events and in the subsequent development of mood disorders. Moreover, this differential vulnerability to stressful events largely depend on the individual’s genotype. In summary, the gene-environment model suggests that the interaction between life stress and genetic vulnerability represents a major risk factor for the development of mood disorders. In fact, the impactful study conducted by Caspi et al. [45] showed that carriers of at least a short allele of the serotonin transporter promoter polymorphism are more likely to display depressive symptoms as compared to individuals homozygous for the long allele. In a similar vein, documents included in cluster #0 mainly focused on the complex between genetic and environmental factors. Although the complex relationship between these factors had been theorized before [89,90], new technological advancements allowed to obtain initial experimental evidence. For instance, some documents showed that stress-induced DNA methylation controls the development of the hypothalamic–pituitary–adrenal axis [101] as well as the corticolimbic circuit of the brain [102,103].

### 4.9. Cluster #8: Circadian Genes

Cluster #8 includes documents published, on average, in 2013. In cluster #8, the major citing documents were authored by Partonen [104] (coverage = 9; GCS = 71), Sjöholm et al. [105] (coverage = 9; GCS = 63), and Soria et al. [106] (coverage = 9; GCS = 267). Several documents in the cluster discussed the role of circadian genes in the etiology of mood disorders [104,107]. Circadian genes belong to the molecular circadian machinery [106]. Genetic variations on circadian genes modulate the brain’s structural development in regions such as the amygdala, the orbitofrontal cortex, and the hippocampus [108]. For their influence on brain development, genetic variations of circadian genes (e.g., CRY1, CLOCK, and NPAS2) are oftentimes associated psychiatric disorders such as anxiety, mood disorders, and alcohol use. With regard to mood disorders, the study by Soria et al. [106] confirmed that specific variations in circadian genes represent a risk factor for the development of mood disorder. Furthermore, Soria et al. [106] suggested that specific variations in circadian genes also determine the polarity of the mood disorder. Specifically, on the one hand, CLOCK and VIP variants appear to be selectively associated with bipolar disorders. On the other hand, CRY1 and NPAS2 appeared to be associated to major depression disorder.

As for the BDNF, circadian genes emerged to be associated with lithium treatment response [109]. Specifically, from the study it emerged that the aryl hydrocarbon receptor nuclear translocator and timeless circadian clock genes are associated with the degree of lithium prophylaxis.

### 4.10. Cluster #12: Mood Disorders and Other Psychiatric Conditions

In cluster #12, the documents were published, on average, in 2013. The major citing documents in cluster #12 were authored by Gomez et al. [110] (coverage = 6; GCS = 12), Green et al. [111] (coverage = 5; GCS = 55), and Amare et al. [112] (coverage = 5; GCS = 98). Documents included in the cluster focused on investigating how common genes that increase the risk of a multitude of psychiatric conditions also increase the risk of mood disorders (e.g., [113]). For instance, Gomez et al. [110] studied the contribution of G72/G30 gene in the 13q32 region in the etiology of mood disorders. This genetic region is of particular interest as it had been previously associated with panic disorder, schizophrenia, and bipolar disorder. Interestingly, after correcting their results, the authors did not find any statistically significant association between the investigated genetic markers and mood disorders. Other documents corroborated the molecular findings for which mood disorders and schizophrenia share part of their genetic structure [114]. Moreover, it emerged that the genetic risk for schizophrenia negatively influences the response to lithium in patients with bipolar disorder [112].

### 4.11. Cluster #1: Genome-Wide Associations Study

Cluster #1 is a group of documents published, on average, in 2015. The major citing documents were authored by Domschke and Reif [115] (coverage = 20; GCS = 28), Barnett and Smoller [116](coverage = 19; GCS = 230), and Fullerton et al. [117] (coverage = 15; GCS = 17). The main thematic focus of documents in cluster #1 is the introduction of GWAS in the genetic research of mood disorders (see, for instance, the impactful document by [54]). As argued by Barnett and Smoller [116], in light of the recent evidence suggesting a polygenetic risk for mood disorders [117], GWAS represent the better option for evaluating the small contributions of several individual genes. GWAS relies on genotyping arrays that assess the whole genome and identify a connection between variants and pathological traits [118]. To help the advancement of GWAS in psychiatry research, studies addressing computational and methodological challenges were included in the cluster. This is the case, for instance, of the original and impactful document by Purcell et al. [49]. In their publication, Purcell et al. [49] introduced PLINK, a tool set to conduct GWAS and population-based linkage analyses. GWAS in mood disorders highlighted the role of neurodevelopmental factors (e.g., nervous system development, cell migration) in the pathogenesis of the disorders [119,120]. Moreover, GWAS corroborate the existence of genetic commonalities between mood disorders, anxiety disorders, and schizophrenia [115,119].

### 4.12. Cluster #13: MicroRNAs

The average year of publication of documents included in cluster #13 is 2015. The major citing documents in cluster #13 were authored by Luoni and Riva [121] (coverage = 22; GCS = 35) and by O’Connor et al. [122] (coverage = 13; GCS = 43). Several documents in the cluster investigated the pivotal role of microRNAs in mood and other psychiatric disorders. MicroRNAs are portion of non-coding RNAs that regulate post-transcriptional gene expression [121,123]. Assessing the contribution of miRNAs in the etiology of mood disorders was made possible by lower costs and advances in analytic methods in molecular genetics. In this way, the sequencing approach (i.e., whole-genome sequencing, whole-exome sequencing) became a valid alternative to the array-based genotyping commonly adopted by GWAS [124]. MicroRNAs regulate crucial biological changes happening during neurodevelopment and in adulthood in the central nervous system, such as neuroplasticity [121,125]. For their role, microRNAs have been suggested to be involved in the pathogenesis of many psychiatric conditions such as schizophrenia and mood disorders [20]. Evidence from animal models shows that chronic exposure to stress reduces the expression of the brain-enriched miRNA-124 in the hippocampus and increases depression-like behaviours [126]. In humans, miR-34a was shown to be deferentially expressed in the anterior cingulate cortex, a crucial structure for mood regulation, of people with major depressive disorder [123]. In animal models, the same microRNA emerged to be a target for mood stabilizers as it regulates levels of metabotropic glutamate receptor 7 [127].

### 4.13. Cluster #4: Polygenic Risk

Cluster #4 includes documents published, on average, in 2020. In cluster #4, the major citing documents were authored by Facal et al. [128] (coverage = 12; GCS = 2), Tubbs et al. [129] (coverage = 12; GCS = 5), and Forstner et al. [8] (coverage = 11; GCS = 1). Thanks to the advancement of genomic analysis (e.g., GWAS) and the investigation of environmental factors, documents in cluster #4 focused on the polygenic risk to general psychopathology and to specific psychiatric disorders [8,130,131]. For this reason, some of the cited documents in the clusters are studies in which GWAS proved useful in clarifying the genetic underpinnings of other psychiatric disorders. For example, the GWAS study on schizophrenia conducted by Pantelis et al. [46], which was one of the most impactful documents in the network, was included in the cluster. By using the polygenic model of mood disorders, researchers were able to integrate the previous findings in more complex etiological models of mood disorders. These models were able to take into account complex factors (e.g., neurotrasmitters, stress response, neurodevelopment, neurogenesis, neurodegeneration) and their regulation at the genetic level of analysis [129]. Once again, with the emphasis on the polygenic nature of mental disorders, boundaries between diagnostic labels became less rigid. For instance, Biere et al. [130] showed that, in young adults, the polygenic risk of bipolar disorders not only predicts a diagnosis of major depressive disorder, but also a diagnosis of attention-deficit/hyperactivity disorder.

### 4.14. Study Strengths and Limitations

The scientometric approach to reviews adopted in the current study proved useful in shading light onto the genetic research of mood disorders. The main strength of the scientometric approach is that it allows to reduce human-related bias in the selection of relevant articles by operating in a data-driven fashion. However, some limitations need to be considered in the interpretation of the current study. First of all, the results depend on the key terms, searching string, and platform chosen for conducting the literature search. Some documents might have been involuntarily neglected because they do not include some of the selected key terms in their title, abstract, or keywords. Similarly, some documents might have been involuntarily excluded because they are not available in Scopus. Another limitation of the study is that the scientometric approach largely depends on quantitative (and not qualitative) relationships between documents. A precious insight can be obtained from the patterns of co-citations. However, it is worth noting that frequent co-citation does not imply the papers’ quality or the citation relationship between the two papers [23]. In fact, some documents might be highly cited because they are groundbreaking, but also because they are highly criticized by other documents in their field. For this reason, in the current study we added a qualitative discussion of the identified network and clusters, as suggested by Hicks et al. [132].

## 5. Conclusions

The current paper adopted a scientometric approach to reviewing in a data-driven way the vast literature on the genetics of mood disorders. A DCA was conducted to identify the most impactful publications and the main thematic domains in the field. Furthermore, a country analysis was performed to identify the most active countries in the literature of interest. Among the 308 documents with a citation burst, the study by Caspi et al. [45] was the most novel and influential. This study was pivotal in the network as it highlighted the importance of considering both environmental and genetic factors when trying to explore the roots of mood disorders. Similarly, authors with affiliations in the United States of America, Belgium, and China were highly impactful in the genetic research on mood disorders. Furthermore, 13 main thematic clusters were identified in the literature of interest. Genetic research in mood disorders was conducted by investigating single gene linkage. However, this approach showed contradictory results in terms of replicability of findings. Subsequently, methodological and statistical advancements allowed to investigate the contribution of neurotransmitter systems, neuroplasticity, and circadian rhythms together with their regulatory mechanisms at the molecular level (i.e., dopamine and serotonin transporter and receptor genes, BDNF, and circadian genes). With the subsequent introduction of the gene-environment framework, epigenetics attracted the interest of the scholars community. In particular, together with coding portions of RNA, the pivotal role played by non-coding portions of RNA (i.e., microRNAs) in regulating neurodevelopment emerged too. However, a clear indication of which environmental factors pose a differential risk for mood disorders and how they do so is still missing in the literature and it might represent material for future investigations. More recently, GWAS allowed to integrate all the aforementioned factors in complex models of psychiatric disorder and led to the conceptualization of the polygenic risk framework. Across clusters, the comparison of the genetic architecture between mood disorders and other psychiatric conditions was a common interest. Genetic similarities between mood disorders and other psychiatric conditions (especially schizophrenia) were supported by findings from molecular studies and GWAS. Another common interest across clusters regarded the interaction between genetic makeup and treatment effectiveness in mood disorders. These initial studies show the premises to provide pharmacological treatments that go beyond the diagnostic label as they are calibrated on the patient’s characteristics (e.g., genome). However, these studies focused uniquely on the genetic profiling for pharmacological treatments. Future studies could extend this approach to investigate the differential response to other types of treatment for mood disorders (e.g., psychotherapy, brain stimulation).

## Figures and Tables

**Table 1:** Summary metrics for the 13 major clusters identified in the DCA network.

Cluster ID	Size	Silhouette	Mean Year	LLR Label	Suggested Label
0	241	0.895	2010	Bipolar disorder	Gene-Environment Interaction
1	232	0.834	2015	Genome-wide association study	Genome-wide association study
2	173	0.907	1999	Bipolar Affective disorder	Methodological refinement
3	150	0.872	2005	Chasing gene	Mood disorders and schizophrenia
4	149	0.941	2020	Polygenic risk score	Polygenic risk
5	117	0.932	2001	Serotonin transporter gene	Serotonin transporter genes
6	85	0.979	1991	Early-onset major Depression	Susceptibility loci for early-onset major depression
8	73	0.943	2013	Therapeutic approaches	Circadian genes
9	68	0.973	1997	Lithium-responsive Affective disorders	Dopamine receptor genes
10	60	0.954	2006	Brain-derived neurotrophic factor	Brain-derived neurotrophic factor
11	56	0.987	1991	Close linkage	Single gene linkage to mood disorders
12	37	0.984	2013	Juvenile-onset major Depression	Mood disorders and other psychiatric conditions
13	28	0.989	2015	Central role	MicroRNAs

**Table 2:** Top 10 documents with highest citation bursts.

Reference	Citation Burstness	Publication Year	Burst Begin	Burst End	Duration (years)	Centrality	Sigma
Caspi et al. [45]	41.14	2003	2004	2011	7	0.067	14.26
Pantelis et al. [46]	29.67	2014	2015	2023	8	0.093	14.08
Barrett et al. [47]	27.31	2005	2008	2013	5	0.006	1.16
Berrettini et al. [50]	26.46	1994	1996	2002	6	0.007	1.21
Stine et al. [48]	25.42	1995	1996	2003	7	0.013	1.38
Straub et al. [51]	23.39	1994	1996	2002	6	0.035	2.22
Lesch et al. [52]	22.87	1996	1998	2004	6	0.017	1.46
Sklar et al. [53]	22.47	2002	2004	2009	5	0.006	1.13
The Wellcome Trust Case Control Consortium [54]	22.26	2007	2008	2013	5	0.018	1.00
Purcell et al. [49]	22.04	2007	2010	2015	5	0.094	7.30

## Data Availability

Not applicable.

## Abbreviations

The following abbreviations are used in this manuscript:

GWAS	Genome-wide associations study
DCA	Document Co-Citation Analysis
PRISMA	Preferred Reporting Items for Systematic Reviews and Meta-Analyses
LLR	Log-Likelihood Ratio
GCS	Global Citing Score
BDNF	Brain-derived neurotrophic factor
